# Extreme Activity of Drug Nanocrystals Coated with A Layer of Non-Covalent Polymers from Self-Assembled Boric Acid

**DOI:** 10.1038/srep38668

**Published:** 2016-12-09

**Authors:** Honglei Zhan, Jun F. Liang

**Affiliations:** 1 Department of Biomedical Engineering, Chemistry, and Biological Sciences, Charles V. Schaefer School of Engineering and Sciences, Stevens Institute of Technology, Hoboken, NJ 07030, USA

## Abstract

Non-covalent polymers have remarkable advantages over synthetic polymers for wide biomedical applications. In this study, non-covalent polymers from self-assembled boric acid were used as the capping reagent to replace synthetic polymers in drug crystallization. Under acidic pH, boric acid self-assembled on the surface of drug nanocrystals to form polymers with network-like structures held together by hydrogen bonds. Coating driven by boric acid self-assembly had negligible effects on drug crystallinity and structure but resulted in drug nanocrystals with excellent dispersion properties that aided in the formation of a more stable suspension. Boric acid coating improved drug stability dramatically by preventing drug molecules from undergoing water hydrolysis in a neutral environment. More importantly, the specific reactivity of orthoboric groups to diols in cell glycocalyx facilitated a rapid cross-membrane translocation of drug nanocrystals, leading to efficient intracellular drug delivery, especially on cancer cells with highly expressed sialic acids. Boric acid coated nanocrystals of camptothecin, an anticancer drug with poor aqueous solubility and stability, demonstrated extreme cytotoxic activity (IC_50_ < 5.0 μg/mL) to cancer cells compared to synthetic polymer coated CPT nanocrystals and free CPT. Surface coating using non-covalent polymers from self-assembled boric acid will have wide biomedical applications especially in biomaterials and drug delivery field.

Camptothecin (CPT) works against a broad spectrum of human cancers including colon, lung, breast, ovarian, and melanoma^1^,^2^,^3^ by acting on topoisomerase I to inhibit DNA replication and RNA transcription processes^4^,^5^,^6^,^7^,^8^. However, clinical trials of CPT formulation have been hampered due to the poor stability and water solubility^9^,^10^. Attempts to efficiently deliver CPT have involved a broad range of drug carriers like micelles, liposome, polymer conjugations, and chitosan complexation^11^,^12^,^13^,^14^; however, all of these methods have drawbacks^15^,^16^,^17^. Recently, various CPT crystals have been successfully prepared on a laboratory scale using different polymers as stabilizing agents. Resulting CPT crystals demonstrated improved solubility and good stability, but only showed similar or slightly better anticancer activity compared to free CPT solution. Studies revealed that different CPT crystals coated by polymers might have decreased cellular uptake, slow dissolution profile, low cancer selectivity, and limited activity against some types of cancer cells^18^,^19^.

Supramolecular chemistry concept has opened the doors for the exploitation of new materials. Although non-covalent polymers are self-assembled small molecules held together by reversible interactions, such as hydrogen bonds, metal-ligand complexation, π–π stacking, and host-guest interactions^20^,^21^, they have a lot in common with their covalent counterpart. Generally speaking, the covalent modifications in polymers are superior to the non-covalent modifications in terms of the stability of the functionalization. However, the covalent modification changes the intrinsic properties of the bioactive monomers; upon melting, they become highly viscous which is the result of entanglement of their macromolecules, limiting their application. On the contrary, the non-covalent polymers are more advantageous in most cases in order to utilize the inherent properties of the bioactive monomers. The high reversibility of the non-covalent bonds ensures that supramolecular polymers are always formed under conditions of thermodynamic equilibrium. The lengths of the chains could be directly tailored via the strength of the non-covalent bond, the concentration of the monomer and the temperature. In addition, other beneficial features of non-covalent polymers include stimuli responsive and self-healing abilities, as well as, dramatic decreases in melt viscosity and permitting facile processing. Consequently, development in this area is clearly inspired by biological systems like drug delivery application. The bioactive monomers could be fine-tuned to meet the exact needs of biological system^22^,^23^,^24^.

The micron-scaled one-dimension structure of boric acid non-covalent polymers is revealed recently^25^. In self-assembled boric acid clusters, one boron atom is surrounded by three oxygen atoms to form triangular BO_3_ groups and hydrogen bonds link the planar BO_3_ groups together^26^. The predicted structures take the form of a petal, boat, bowl, cage, or tube, all of which prove boric acid clusters to behave similarly to the well-studied carbon clusters^27^. In this study, we used non-covalent polymer of boric acid as the capping agent to stabilize drug nanocrystals. We found that boric acid supported the formation of stable and nano-sized drug crystals but had negligible effects on drug crystallinity and structure. Resulting drug nanocrystals showed excellent dispersion properties, greatly improved drug stability, cancer selectivity, and demonstrated extreme anticancer activity when the free drug was not active at all. Most importantly, in comparison with the traditional polymer coated CPT nanocrystals, this novel formulation displayed advanced physio-chemical properties and especially advanced anti-cancer activity. In addition, the non-covalent polymer preparation process is facile and green, which makes CPT nanocrystal formulation feasible and more favorable in clinical practice.

## Results

### Preparation of CPT nanocrystals coated by non-covalent polymer of boric acids

Nano-sized CPT crystals were successfully prepared in pH = 4 water solutions containing 0.00, 0.25, 0.50, and 1.00% of boric acid. CPT crystals prepared in the presence of boric acid had gradually increased Zeta potentials (Table 1). Since boric acid is a weak acid in pure water, these boric acid concentration-dependent Zeta potential changes reflected the absorption of boric acid on CPT nanocrystal surfaces. Stabilized Zeta potentials were observed when boric acid concentration reached 1.00% (w/v), implying that adsorbed boric acid on nanocrystal surfaces reached equilibrium and formed stable structures. In addition, great Zeta potential value fluctuations (batch-to-batch as well as during storage) were observed in nanocrystals prepared in low concentrations of boric acid. Such a phenomenon might result from the adsorption and de-adsorption dynamics of free form boric acid on CPT crystal surfaces before they reached certain concentrations to form non-covalent polymers. In a control study, the same hydroxyl-group rich but covalently linked polymer, poly (vinyl alcohol) (PVA), was used as the stabilizing agent. CPT crystals prepared in 1.00% PVA and 1.00% boric acid had close Zeta potential values, supporting the formation of non-covalent boric acid polymers. However, compared to 1.00% boric acid coated CPT nanocrystals, named as BA-coated CPT nanocrystals in this study, 1.00% PVA coated CPT crystals had bigger sizes. We know that stabilizers in the form of either surfactants or polymers are required in size-reduction processes to mask the high-energy surfaces of small sized particles. The former provides electrostatic barriers to aggregation and the absolute value of Zeta potential should be at least 30 mV, whereas the latter provides steric barriers. Steric stabilization is superior to surfactants and only a minimum layer thickness is required^28^,^29^,^30^. The small crystal size and Zeta potential value also suggest that boric acid stabilized the nanocrystals in the form of non-covalent polymers rather than in the form of surfactant (free boric acid). Moreover, 1.00% BA-coated CPT nanocrystals displayed narrow size distribution in terms of the lowest PdI values (Table 1).

**Figure 1:**
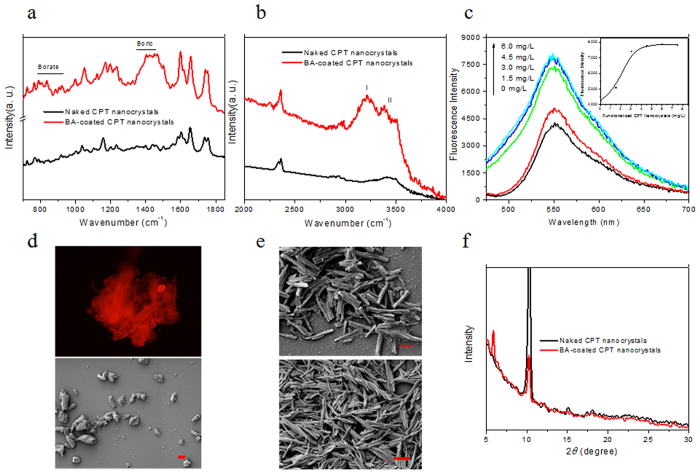
(**a**,**b**) ATR-FTIR spectra of naked and BA-coated CPT nanocrystals. (**c**) Reaction of orthoboric groups on BA-coated CPT nanocrystals to ARS caused ARS emission spectrum change. Insert, a plot of fluorescence intensity of ARS emission peak at 550 nm against the concentrations of BA-coated CPT nanocrystals. (**d**) Fluorescence images of boric acid polymer layer stained by ARS (Top), SEM images of boric acid polymer layer on surface of CPT nanocrystals (Bottom). (**e**) SEM images of naked (Top), BA-coated (Bottom) CPT nanocrystals. Scale bars = 100 nm. (**f**) X-ray diffraction patterns of naked and BA-coated CPT nanocrystals.

Formation of non-covalent boric acid polymers on CPT nanocrystals was confirmed using ATR-FTIR. Simultaneous appearance of two groups of B-O stretching bands at 850~1050 cm^**−***1*^ (borate, from self-assembled or polymerized boric acid) and 1300~1450 cm^**−**1^ (boric, free form of boric acid)^31^ were observed in BA-coated CPT nanocrystals (Fig. 1a). It should be noted that ATR-FTIR spectra of BA-coated CPT nanocrystals showed at least two types of hydrogen bonds in the ranges of 3000~3250 cm^−1^ and 3300~3500 cm^−1^ (Fig. 1b). Following an established method^32^, we could easily come to the conclusion that peaks at 3000~3250 cm^−1^ represented the bridge between the OH groups of boric acid (-B-OH….HO-B-) while peaks at 3300~3500 cm^−1^ were from adsorbed atmospheric water to boric acid (-B-OH….HOH) on CPT crystal surfaces. The highest intensity of peak at 3000~3250 cm^−1^ confirmed that majority of boric acid on BA-coated CPT nanocrystals were in the crystal form and existed as polymers with network-like structures.

The amount of boric acid polymer on CPT nanocrystal surfaces was quantified through an ARS-based spectrophotometer assay. ARS reacted with boric acid on CPT nanocrystal surfaces resulting in increased fluorescence intensity and emission peak shifts (Fig. 1c). Increased fluorescence intensity had a linear correlation with boric acid concentration on CPT nanocrystals. Based on the plot of fluorescence intensity and crystal concentrations (Fig. 1c, insert), we estimated that 1 mg of CPT crystals was coated with approximately 1 mg of non-covalent boric acid polymers. To further prove the formation of boric acid polymers, we dissolved CPT in BA-coated CPT nanocrystals using organic solvents. As CPT in nanocrystals was extracted, polymer capsules, with comparable nano-scaled sizes to CPT nanocrystals, were observed (Fig. 1d, bottom). However, due to the removal of CPT crystals, the hollow boric acid polymer coatings were unable to hold the original sizes and thus showed shrunk and collapsed structures. We then used Alizarin Red S (ARS), a fluorescence dye, which can react with orthoboric groups specifically^33^, to stain boric acid coatings. Hollow boric acid polymer capsules left after CPT extraction became visible under confocal microscopy (Fig. 1d, Top).

It was interesting to note that although BA-coated CPT nanocrystals had the same rod-like structure as naked CPT nanocrystals (Fig. 1e), they gave altered X-ray diffraction patterns (Fig. 1f). Besides characterized peaks of CPT nanocrystals at 8.8°, 10.2°, 11.8°, 14.8° and 18.2°, BA-coated CPT nanocrystals showed three distinct peaks at 5.8°, 15.2°, and 28.0°. Peaks at 15.2° and 28.0° were typical peaks for boric acid^34^ while the peak at 5.8° represented another form of CPT crystals occurred under varied crystallization conditions^33^.

### Properties of BA-coated CPT nanocrystal suspensions

**Figure 2:**
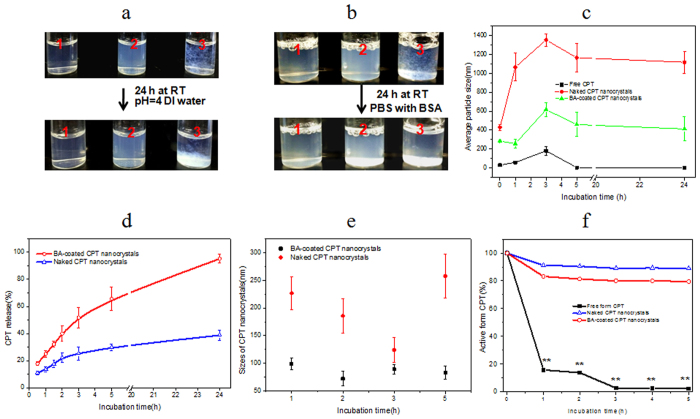
(**a**,**b**) Solubility test: Images of suspension from BA-coated CPT nanocrystals (1), naked CPT nanocrystals (2), and free CPT (3) immediately after preparation (top) or left at room temperature for 24 hours (bottom), the dispersant is pH = 4 DI water (**a**) or 50 mg/mL BSA/PBS solutions (**b**). (**c**) Size changes of free CPT, naked and BA-coated CPT nanocrystals at different time intervals after preparation (pH = 4, DI water). (**d**) *In vitro* dissolution dynamics of naked and BA-coated CPT nanocrystals. Experiments were conducted in solutions of physiological conditions (PBS, pH = 7.4). (**e**) Size changes of naked and BA-coated CPT nanocrystals under physiological conditions (PBS, pH = 7.4). (**f**) Deactivation dynamics of free form CPT and CPT nanocrystals under physiological conditions (PBS, pH = 7.4). **P < 0.01, BA-coated CPT nanocrystals *vs* free form CPT.

Because of the poor water solubility of CPT, direct addition of CPT into aqueous solution (pH = 4 DI water) led to incompletely dissolved, cloudy, and unstable suspensions (Fig. 2a). CPT was quickly precipitated at the bottom of containers. Due to the small sizes, CPT nanocrystals could be easily dispersed in aqueous solutions to form stable suspensions. However, unlike the milk-like suspensions of naked CPT nanocrystals, suspensions of BA-coated CPT nanocrystals were clear and nearly transparent. No visible precipitation was observed even in solutions with high ionic strength and high concentration of proteins (albumin, 50 mg/mL) (Fig. 2b). Stability of CPT solution and two CPT nanocrystal suspensions, in terms of particle sizes, were monitored over the period of 24 h incubation (Fig. 2c). Sizes of naked CPT nanocrystals experienced remarkable changes over the time, which could be explained by Ostwald ripening phenomenon, that is, small particles are dissolved and molecules are re-deposited to larger particles. On the contrary, because boric acid polymers served as stabilizing agents, BA-coated CPT nanocrystals maintained sizes in nanometer ranges with slight variations.

Besides poor solubility, free CPT underwent water hydrolysis and became deactivated rapidly under physiological conditions, forming inactive CPT via lactone ring opening process^9^. In pH = 7.4 PBS solution, nearly 90% of free CPT was converted into the inactive form after 1.0 hour of incubation (Fig. 2f). Crystallization prevented CPT from aqueous hydrolysis and thus CPT nanocrystals had good chemical stability. It was interesting to notice that naked CPT crystals dissolved slowly under physiological conditions and less than 30% of the total free form CPT was released after 5 hours incubation (Fig. 2d). Further studies revealed that naked CPT nanocrystals showed an irregular but continuous changing of crystal sizes, indicating a dynamic process between dissolution and aggregation (Fig. 2e). Due to the rapid release at the early phase (0–3 h), dissolution in naked CPT nanocrystals was predominantly taking place (Fig. 2d). Rapid dissolution of some CPT in pre-formed crystal aggregates (naked CPT crystals are unstable due to Ostwald ripening phenomenon) would lead to the collapse of crystal aggregation and thus re-dispersion of naked CPT crystals in solutions. However, as more CPT dissolved, CPT crystal dissolution entered into a slow phase (3~24 h, Fig. 2d) and instead, collision-induced crystal aggregation occurred. Therefore, increased crystal sizes were observed after the slow phase (Fig. 2e). On the contrary, BA-coated CPT crystals showed enhanced dissolution dynamics, and nearly 70% of total CPT was dissolved from nanocrystals after 5 hours of incubation (Fig. 2d). The large difference in dissolution kinetics between the two nanocrystals remained even after 24 h incubation (95.2% *vs* 38.8%). Excellent dispersion helped BA-coated CPT crystals dissolve quickly to release free CPT. In addition, BA-coated CPT nanocrystals maintained unchanged crystal sizes during the entire period of dissolution (Fig. 2e), and indicating that crystal aggregation did not occur and dissolution was the only direction. This uni-modal model was attributed to the good dispersion and robust dissolution properties of BA-coated CPT nanocrystals according to boric acid non-covalent polymer coating.

### Cross-membrane transportation and cell uptakes of BA-coated CPT nanocrystals

**Figure 3:**
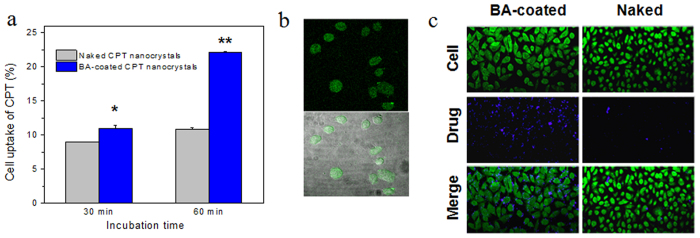
(**a**) Intracellular CPT concentration changes over incubation time. To be specific, A549 cells were exposed to naked or BA-coated CPT nanocrystals for the indicated time and intracellular CPT was extracted at the end of incubation and then quantified. Uptake rates were expressed as intracellular CPT *vs* total CPT loaded in each cell culture well. *P < 0.05, **P < 0.01. (**b**) Cell uptake images from A549 cells exposed to FITC labeled BA-coated CPT nanocrystals. Top, confocal microscopy image; Bottom, confocal microscopy image was merged with contrast microscopy image of cells. Incubation time = 45 min. (**c**) Confocal images of uptake of BA-coated CPT nanocrystals and naked CPT nanocrystals (blue fluorescence) on A549 cells (green fluorescence) for 1 h incubation.

A major advantage of drug nanocrystals is that cells can absorb nano-sized matters by engulfing them directly, an energy-dependent process called endocytosis^35^. However, BA-coated CPT nanocrystals showed dramatically increased cell uptake rates in comparison with naked CPT nanocrystals (24% *vs* 10%, **P < 0.01) when incubated for 60 min (Fig. 3a). We know that orthoboric groups have specific reactivity to diols^36^. Boric acid and many of its derivatives can react with sugar moieties on cell surfaces to demonstrate good cell permeability^37^,^38^. The time-dependent cell uptake profile (Fig. 3) confirmed the reaction between cells and BA-coated CPT nanocrystals. Therefore, the active cross-membrane transportation mechanism enabled BA-coated CPT nanocrystals to enter cells more efficiently.

### Extreme activity and cancer cell line selectivity of BA-coated CPT nanocrystals

We compared the anticancer activity of BA-coated CPT crystals with free CPT, naked CPT nanocrystals, and PVA-coated CPT nanocrystals (covalent polymer coated formulation, as a control group). Since it was impossible to prepare free CPT solution higher than 45 μg/mL without bulky precipitation, for fair comparison, cytotoxicity tests were performed in a CPT concentration range of 0~45 μg/mL. In addition, for CPT nanocrystals, cell uptake and release of free CPT occurred simultaneously during incubation. Therefore, we performed cytotoxicity tests at two time points by exposing cells to drugs for 5 and 24 hours, respectively. *In vitro* cytotoxicity results from 5 hours incubation mainly reflected the contribution of BA-coated CPT nanocrystals which were already taken in by cells.

**Figure 4:**
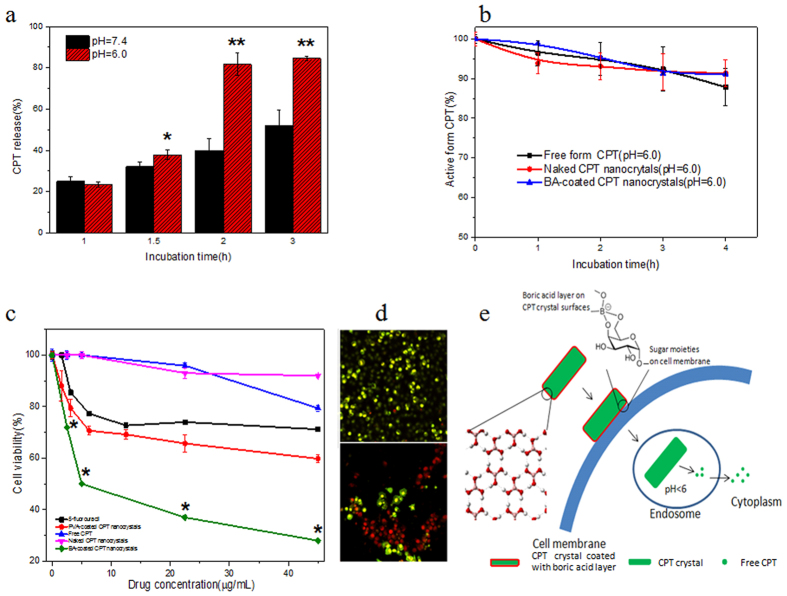
(**a**) Comparison of CPT release ratio from BA-coated CPT nanocrystals at physiological and mild acidic conditions. *P < 0.05, **P < 0.01. (**b**) Deactivation dynamics of free form CPT and CPT nanocrystals under tumor microenvironment (PBS, pH = 6.0). (**c**) Anticancer activity of BA-coated CPT nanocrystals in comparison with free CPT, naked CPT nanocrystals, PVA-coated CPT nanocrystals and anticancer drug 5-fluorouracil against A549 cells at 37 °C(n = 6). Data were expressed as mean ± SD. **P < 0.01, naked CPT nanocrystals *vs* BA-coated CPT nanocrystals. (**d**) Cells stained using LIVE/DEAD kit after treatment with naked (top) and BA-coated (bottom) CPT nanocrystals, respectively. Experiments were performed on human lung carcinoma A549 cells. Drug-cell incubation time was 5 h. (**e**) Mechanistic illustration of the extreme anticancer activity of BA-coated CPT crystals.

Free CPT, naked CPT and PVA-coated CPT nanocrystals showed limited cytotoxic activity to cancer cells after 5 hours incubation (Fig. 4c). In both cases (Free CPT and naked CPT nanocrystals), nearly 90% of cancer cells were still alive even at high CPT concentrations. The results were consistent with previous publications^18^,^39^. To be specific, the low solubility of crude CPT (1.2 μg/mL), the poor dispersion property, and the rapid hydrolysis rate (Fig. 2a,b and f), all contribute to the inefficient anti-cancer activity of free CPT. In contrast, BA-coated CPT nanocrystals demonstrated extreme anticancer activity with an IC_50_ value of less than 5.0 μg/mL. The overall anti-cancer activity of BA-coated CPT nanocrystals was more potent than that of 5-fluorouracil (*P < 0.05), a common clinical anticancer drug. In addition, it should be noted that A549 cells showed a typical resistant response to 5-fluorouracil, and further improved anticancer activity was not observed when 5-fluorouracil concentration reached 10 μg/mL and above. This is a common phenomenon in today’s chemotherapy with widely spread drug resistance effects from tumors. Most treatments using single anticancer drugs over time have very limited therapeutic efficacy^40^ due to the drug resistance effect. It is well known that tumor cells exposed to chemotherapeutic agents over time may develop, both *in vivo* and *in vitro*, a multidrug resistant (MDR) phenotype, which is generally associated with over-expression of P-glycoprotein (P-gp), the family of multidrug resistance-associated proteins (MRPs) and lung resistance-related protein (LRP)^41^. A549 cells have extensively proved to exhibit an intrinsic multidrug resistant phenotype with moderate expression level of P-gp, functional MRP1, and over expression of LRP. LRP proved to be a critical component of a pathway inducing multidrug resistance^41^,^42^,^43^. However, BA-coated CPT nanocrystals gave a nice dose-dependent curve.

The extreme anti-cancer activity of BA-coated CPT nanocrystals was confirmed on the experiment with extended (24 hours) drug-cell incubation time when the cytotoxcity of free CPT and naked CPT nanocrystals became measurable. Compared to free CPT, naked and PVA-coated CPT nanocrystals showed mildly (2.6 and 5.7 times) improved cytotoxicity while BA-coated CPT nanocrystals demonstrated 65 times increased activity (*P < 0.05) (Table 2).

### Cell selectivity and anti-cancer mechanism study

We compared cytotoxicity of BA-coated CPT nanocrystals with free CPT on selected normal (SC and NHDF) and cancer (Hela and A549) cells, and found that BA-coated CPT nanocrystals had dramatically increased activity to cancer cells (*P < 0.05) while their activity to two normal cells kept unchanged (Table 3), implying cancer cell selectivity. To confirm and explain these results, we extended selectivity tests to glyco-engineered cells with varied sialic acid expressions. By adding N-acetylmannosamine analogue, Ac_4_ManNAc^44^,^45^, to the cell culture medium, we increased sialic acid expression on the membrane of SC cells by nearly three times (Table 4). Interestingly, although normal SC cells responded to free CPT and BA-coated CPT nanocrystal treatment to the same extent, they became more sensitive to BA-coated CPT nanocrystals when high concentrations of sialic acid were expressed on cell surfaces. To confirm the role of orthoboric group reactions with glycocalyx in the extreme anti-cancer activity of BA-coated CPT nanocrystals, a competitive study using free boric acid was conducted on SC cells with manipulated high expression of sialic acid. We found that the cytotoxicity of BA-coated CPT nanocrystals decreased in the presence of boric acid (Table 4) and complied in a dose-dependent manner, that is, liner to the concentrations of boric acid added. These results provided additional supports to the cell targeting and membrane translocation roles of boric acid coatings (Fig. 3) and explained the cancer cell selectivity (Table 3) of BA-coated CPT nanocrystals.

## Discussion

Crystallization is a new formulation technology for drugs with poor solubility^46^. Despite different approaches such as milling, precipitation, and homogenization^47^, crystallization results in micro- and nano-sized crystals that prefer to aggregate due to significantly increased Gibbs free energy. In practice, carefully selected capping agents, known as stabilizers, are required in order to offer high drug loading, tolerance, and stability^48^. Currently, various polymeric surfactants are used as stabilizers to prevent Ostwald’s ripening phenomena^49^. Aside from the safety issues associated with clinical trials, these macromolecules complicate drug crystal formulation considerably and may make them difficult to prepare nano-sized drug crystals.

We tried for the first time to use non-covalent polymers of boric acid as the capping agent to stabilize drug crystals. Results from both theoretical predictions^27^ and experiments^25^ confirmed that acidic pH (pH < 5), temperature (10~30 °C), and concentrations (0.1~2.0%) were critical for boric acid to self-assemble into non-covalent polymers. Meanwhile, acidic (pH = 4) and room temperature were also optimal crystallization conditions for CPT^18^,^39^. CPT formed the most stable and smallest size of nanocrystals in the presence of 1.0% boric acid. CPT nanocrystals prepared at low concentrations (<1.0%) of boric acid contained non-polymerized (i.e. free boric acid) boric acid on crystal surfaces, and thus had low stability and high negative zeta potentials (Table 1). Although boric acid has low toxicology profile^50^,^51^, high concentrations of boric acid can affect tubular polymerization and proteasome^51^,^52^,^53^,^54^. Therefore, to ensure the clinical significance of our studies, we avoided CPT formulation with high concentration (>1.0%) of boric acid. Boric acid at corresponding concentrations as in BA-coated CPT nanocrystals was not cytotoxic at all (data not shown).

Due to the large surface area on nano-sized crystals, significant amount of boric acid were deposited on CPT nanocrystals. However, BA-coated CPT crystals were able to maintain similar structure and crystallinity as that of naked CPT nanocrystals (Fig. 1e) because of non-covalent and self-assembly natures of boric acid coatings. A boric acid layer reduced the Gibbs free energy of CPT nanocrystals and thus prevented them from forming dynamic aggregate in solutions (Fig. 2a,b,c,e). In addition, the hydrophilic property of boric acid and small sizes also helped BA-coated CPT nanocrystals form stable suspensions (Fig. 2a,b). The good solution dispersion and lower possibility of crystal aggregation also explained the rapid release profile of BA-coated CPT nanocrystals (Fig. 2d,e).

We know that orthoboric groups can react with diols specifically^36^. Therefore, boric acid and many of its derivatives can cross cell membranes actively and show good cell permeability^37^,^38^ because of their reactions with diols in cell glycocalyx^55^,^56^. BA-coated CPT nanocrystals exhibited much better cell uptake rates in comparison with naked CPT nanocrystals (Fig. 3). Although mammalian cells are covered by a dense layer of glycol conjugates^57^, cancer cells frequently display high levels of glycans with fundamentally different structures from normal cells^58^,^59^. Highly expressed sialic acids (sias) are found in carcinomas of the colon, breast, cervix, choriocarcinomas, and acute myeloid leukemias^59^,^60^, and often correlates with poor prognosis of many human malignancies^58^. We have found that BA-coated CPT nanocrystals were more active to engineered cells with high sialic acid expressions (Table 4), confirming their selectivity to cancer cells (Table 3).

Besides improved solubility, nanocrystal formulation has another advantage for CPT since it can prevent CPT from rapid hydrolysis in aqueous solution. In addition, because cells can absorb nano-sized matters by engulfing them directly, an energy-dependent process called endocytosis^35^. Despite these advantages, both PVA-coated and naked CPT nanocrystals only showed mildly increased (5.7 and 2.6 times) anticancer activity in comparison with free CPT (Fig. 4c, Table 2), which explained varied anticancer activities of CPT nanocrystals in different publications^18^,^39^. However, improved anticancer activity in BA-coated CPT nanocrystals was remarkable (41~65 times on A549 cells) (Tables 2 and 3). Since naked and BA-coated CPT nanocrystals had the same preserved activity (Figs 2f and 4b), moderately increased cell uptake (24% *vs* 10%) was unable to explain the extreme cytotoxicity of BA-coated CPT nanocrystals. Because of the non-covalent polymer nature of boric acid coatings, BA-coated CPT nanocrystals had a rapid release profile (Fig. 2d) which allowed the quick accumulation of free CPT in cytoplasm. In fact, we also found that BA-coated CPT nanocrystals had a pH-dependent release profile with a dramatically accelerated release rate at mild acidic pH (Fig. 4a). Since intracellular trafficking of drug crystals might encounter numerous acidic microenvironments with pHs = 5.5~6.5^61^, rapid and timely release would ensure quick accumulations of free CPT inside cancer cells to reach the minimal lethal concentration and cause cell death. It was reported that CPT in solutions was stable when pH < 5.5 and became inactive when pH > 9 after short periods of incubation^37^. Under the mild acidic pH of a tumor environment, even free form CPT could maintain about 90% activity after 4 h incubation (Fig. 4b). Such results confirm the activity of CPT released from BA-coated CPT nanocrystals at tumor sites. Apparently, the extreme anticancer activity of BA-coated CPT nanocrystals attributed to its advanced features of good dispersion ability, active membrane translocation, and rapid dissolution and intracellular release. A possible acting mechanism of BA-coated CPT nanocrystals was proposed and illustrated in Fig. 4e. This is the first study to prove that the advantages of non-covalent polymers surpass those of conventional polymers in nanocrystal coating for drug delivery.

## Methods

### Materials

Boric acid granule was purchased from Baker Chemical Company (Phillipsburg, NJ, USA). Cells and cell culture related products were purchased from Invitrogen (Carlsbad, CA, USA). Nucleopore polycarbonate membranes (50 nm in size) were purchased from Whatman Company. Camptothecin powders (purity > 99%), Polyvinyl alcohol (PVA), Alizarin Red S(ARS), Albumin, bovine serum (BSA) and all other chemicals were purchased from Sigma-Aldrich Company (St. Louis, MO, USA). Tetra-Oacetyl-2-acetamido-2-deoxy-beta-D-mannose (Ac_4_ManNAc) was a gift from New Zealand Pharmaceuticals Ltd. (Palmerston North, New Zealand).

### Preparation of CPT nanocrystals coated by boric acid

An anti-solvent precipitation augmented by sonication^18^ was used. CPT (1.0 mg) dissolved in 200 μL DMSO was injected into 10 mL pH = 4 water solution with different concentrations (0.0~1.0%, w/v) of boric acid. The mixture was irritated by intense sonication followed by rapid stirring (500 rpm for 10 minutes, 300 rpm for 30 minutes, and 100 rpm for several hours) at room temperature for boric acid self-assembly and polymerization. Reactants and organic solvent were removed through filtration using polycarbonate membranes. CPT crystals formed in the absence or presence of boric acid were named as “naked” or “BA-coated” CPT nanocrystals, respectively. In addition, CPT crystals formed in the presence of synthetic covalent polymer PVA (1.0%, m/v) was used for comparison.

The crystallographic patterns of CPT nanocrystals (Naked and BA-coated) were studied using an X-ray Diffractometer (Rigaku, Tokyo, Japan) by scanning from 5° to 30°. A Bragg angle of *2θ* was recorded at a speed of 0.03°/min^19^,^62^. Crystal sizes, the polydispersity index (PdI) and surface potentials of CPT crystals (Naked, BA-coated and PVA-coated CPT nanocrystals) were measured using Zeta nanosizer (Malvern Instruments Ltd.)^44^. Freshly prepared CPT nanocrystals in pH = 4 solutions were subjected to a brief (60 seconds) sonication before measurement. Surface morphology of CPT crystals (Naked, BA-coated) was studied using an established scanning electron microscopy (SEM) analysis^63^. SEM images of CPT crystals were acquired at Auriga Modular Cross Beam workstation (Carl Zeiss, Inc.).

### Analysis and quantification of boric acid self-assembly and polymerization

The presence of self-assembled boric acid on CPT crystals and formation of non-covalent boric acid polymer were further analyzed using ATR-FTIR, confocal microscopy, fluorescence spectrophotometer, and SEM imaging. ATR-FTIR spectra of CPT nanocrystals (Naked, BA-coated) were recorded on a Bruker Instruments Tensor 27 FTIR spectrometer. Experiments were set to a resolution of 4 cm^−1^ and a total of 32 scans per sample^64^. Opus software was used to collect and analyze the data. For confocal microscopy and fluorescence spectrophotometric assay, BA-coated CPT nanocrystals were incubated with Alizarin Red S (ARS) to allow the orthoboric groups to react to form an ARS-boric acid fluorescent complex^65^,^66^. The emission spectra of ARS-boric acid were recorded using a fluorescence microplate reader (Biotek Inc.) by setting excitation wavelength at 485 nm. The amount of boric acid on CPT crystal surfaces was estimated against a calibration curve prepared from free boric acid solution (0~50 μg/mL) with a fixed concentration of ARS (10^−4^ M, pH = 7.4). In addition, BA-coated CPT nanocrystals suspension was first loaded onto the eight-chamber tissue culture glass slide and then air-dried with nitrogen. DMSO was then added into the well several times to dissolve and extract CPT. Resulting boric acid polymer capsules were stained by ARS and visualized using a Zeiss LSM510 Confocal Microscope (Carl Zeiss Inc., Thornwood, NY). In the SEM assay, boric acid polymer capsules left after CPT extraction were air-dried and then sputter coated with a conductive layer of gold palladium (Au/Pd) for 1.0 min. SEM images were acquired at Auriga Modular Cross Beam workstation (Carl Zeiss, Inc.).

### Dispersion, dissolution, and stability studies

Free CPT, naked CPT nanocrystals and BA-coated CPT nanocrystals were transferred into DI water (pH = 4) or PBS/BSA solution (pH = 7.4, BSA concentration was 50 mg/mL) with a final CPT concentration of 50 μM. At the specific time point, an aliquot of suspension was sampled for solubility or dispersion study. Size changes of different drug formulations in pH = 4 DI water were monitored over the time of 24 h incubation.

Crystal suspensions were prepared by adding CPT nanocrystals into pH = 7.4 PBS. Nanocrystal suspensions were incubated at 37 °C under constant shaking. Small amounts of suspension were withdrawn at pre-determined time intervals to examine crystal dispersion, dissolution, and size changes. In the dissolution assay, collected crystal suspensions were forced to pass through polycarbonate membrane. Free CPT released from crystals was quantified using UV spectrophotometric assay against a calibration curve.

Although CPT is stable in an acidic environment of pH = 4, the lactone ring can undergo an opening at physiological pH and can be converted into an inactive carboxylate form. An established fluorescence assay was applied to monitor the conversion process of CPT from an active form (lactone) to an inactive (carboxylate) form^67^. CPT solutions prepared in pH = 4 and pH = 10 environment were used as 100% active and 100% inactive control groups, respectively. In addition, to examine the effect of tumor microenvironment on drug stability and release profile, related assays were also performed in PBS at pH = 6.0.

### Cellular uptake analysis

Cell uptake was conducted by incubating CPT crystals with human lung carcinoma A549 cells cultured on 24-well plates at 37 °C. At the end of different time points, cells were washed with cold PBS three times to remove loosely bound CPT crystals on cell surfaces. CPT inside cells was extracted using DMSO and then quantified using a spectrophotometer^18^.

To further visualize the uptake of BA-coated CPT nanocrystals, a separate experiment was conducted. BA-coated CPT nanocrystals were labeled by fluoroscein isothiocyanate (FITC) via non-covalent interactions. To be specific, 1 mL 0.5 mg/mL FITC solution in PBS (pH = 4) was mixed with 2 mg BA-coated CPT nanocrystals. Because of the low reactivity of FITC with hydroxyl groups^68^, the mixture was stirred overnight in the dark at room temperature. The FITC labeled CPT nanocrystals were collected by centrifugation (10 min, 10,000 rpm, 25 °C) and washed with PBS. Cellular uptake of FITC conjugated BA-coated CPT nanocrystals was conducted on human lung carcinoma A549 cells grown on collagen-coated eight-chamber tissue culture glass slide (BD Falcon, Bedford, MA, USA) and the drug incubation time was 45 min. FITC-labeled BA coated CPT nanocrystals inside cells (green fluorescence, FITC channel) were visualized under confocal microscope (Carl Zeiss Inc., Thornwood, NY) by setting excitation wavelength at 488 nm.

In addition, to observe accumulation and localization of total drug (Free form, intact nanocrystals or an intermediate state), A549 cells were seeded in eight-chamber tissue culture glass slide (BD Falcon, Bedford, MA, USA) at a density of 5.0 × 10^4^ cells per well and incubated overnight. The cells were washed by PBS and then incubated with BA-coated CPT nanocrystals and naked CPT nanocrystals, respectively. The total CPT concentration was set as 50 μM. After 1 hour, the cells were thoroughly rinsed with PBS three times to eliminate any excess of drug, and then fixed in 4% paraformaldehyde for 15 min; the cells were also stained by Syto9 dye. Confocal images were acquired under a confocal microscope using the DAPI channel (total CPT, blue fluorescence) and Syto9 channel (live cells, green fluorescence) (Carl Zeiss Inc., Thornwood, NY). The accumulation and localization of CPT (blue fluorescence) were visualized.

### The activity and selectivity of BA-coated CPT nanocrystals

The *in vitro* cytotoxicity assay was conducted on selected cell lines including human lung carcinoma A549 cells, human cervical carcinoma Hela cells, normal human dermal fibroblast NHDF cells, normal mice Schwann SC cells. Cells were seeded in a 96-well plate at a density of 5 × 10^3^/well and cultured in the proper media (F12K for A549, MEM for Hela, DMEM for NHDF and SC cell lines) supplemented with 10% fetal bovine serum at 37 °C. Free CPT, CPT nanocrystal suspensions (Naked, BA-coated, PVA-coated), and 5-fluorouracil of various concentrations were introduced during culture media change after overnight culture. Cell viability was determined using MTT assay after 5 h or 24 h incubation^69^. Statistical significance was determined by Student’s t test^70^.

### SC cells with varied sialic acid expression

An established metabolic glycoengineering approach using metabolic monosaccharide^44^ was used. Normal Schwann SC cells were cultured in the presence of 100 μM Ac_4_ManAc for 48 hours to acquire sialic acid high expression SC cells. *In vitro* cytotoxicity assays of BA-coated CPT nanocrystals and free CPT were conducted on the normal SC cells and sialic acid high expression SC cells, respectively. In addition, a competitive cytotoxic assay was also conducted on SC cells with highly expressed sialic acid using BA-coated CPT nanocrystals in the presence of different concentrations (5 μg/mL and 45 μg/mL) of free boric acid. Cell viability was determined using MTT assay after 5 h incubation.

### Statistical analysis

Results were presented as the mean ± standard deviation. The statistical significance of the results was analyzed using the two-tailed independent sample t test. Statistical significance was represented by *P < 0.05 and **P < 0.01.

## Additional Information

**How to cite this article**: Zhan, H. and Liang, J. F. Extreme Activity of Drug Nanocrystals Coated with A Layer of Non-Covalent Polymers from Self-Assembled Boric Acid. *Sci. Rep.* **6**, 38668; doi: 10.1038/srep38668 (2016).

**Publisher's note:** Springer Nature remains neutral with regard to jurisdictional claims in published maps and institutional affiliations.

## Figures and Tables

**Table 1:** Properties of CPT crystals prepared in the presence of boric acid and PVA polymer.

Concentration (w/v)%	Crystal size (nm)	PdI	Zeta potential (mV)
*Boric acid coated*			
0.00	429.2	0.412	−11.4
0.25	204.4	0.197	−40.1
0.50	268.8	0.202	−21.5
1.00	283.0	0.172	−8.27
*Poly(vinyl alcohol*) (*PVA) coated*			
1.00	496.0	0.586	−5.82

**Table 2:** Cytotoxicity of free CPT or different CPT nanocrystals against A549 cells after 24-hour-incubation.

	Free CPT	Naked CPT nanocrystals	PVA-coated CPT nanocrystals	BA-coated CPT nanocrystals
IC_50_ (μg/mL)	183	70.9	32	2.8
Activity increase (fold)^a^		2.6	5.7	65.4

^a^In comparison with free CPT.

**Table 3:** Cytotoxicity (IC_50_, μg/mL) of CPT nanocrystals on different cell types after 5-hour-incubation.

Cells	Free CPT	BA-coated CPT NC	Activity increase
A549 (Cancer)	>250	6	>41
Hela (Cancer)	>250	26	>9
NHDF (Normal)	>250	>250	N/A
SC (Normal)	>250	>250	N/A

**Table 4:** Cytotoxicity comparison on SC cells with normal or elevated expression of sialic acid.

	Sialic acid expression (μM/10^7^ cells)	IC_50_ value (μg/ml)		Activity increase^a^ (fold)
		Free CPT	BA-coated CPT NC	
Untreated	0.39 ± 0.05	>250	>250**	No change
Ac_4_ManNAc treated	1.12 ± 0.11	>250	59	>4.2
Ac_4_ManNAc treated in the presence of 5 μg/mL of free boric acid	1.12 ± 0.11	>250	77*	>3.2
Ac_4_ManNAc treated in the presence of 45 μg/mL free boric acid	1.12 ± 0.11	>250	>250*	No change

^a^In comparison with free CPT; ^*^P < 0.05, ^**^P < 0.01, compared with Ac_4_ManNAc treated group.
